# Medication Management: The Macrocognitive Workflow of Older Adults With Heart Failure

**DOI:** 10.2196/humanfactors.6338

**Published:** 2016-10-12

**Authors:** Robin S Mickelson, Kim M Unertl, Richard J Holden

**Affiliations:** ^1^ Vanderbilt School of Nursing, Vanderbilt University The Center for Research and Innovation in Systems Safety (CRISS) Vanderbilt University Medical Center Nashville, TN United States; ^2^ The Center for Research and Innovation in Systems Safety (CRISS) Vanderbilt University Medical Center Nashville, TN United States; ^3^ Department of Biomedical Informatics Vanderbilt University School of Medicine Nashville, TN United States; ^4^ Department of BioHealth Informatics Indiana University School of Informatics and Computing Indianapolis, IN United States

**Keywords:** aged, medication therapy management, medication adherence, workflow, cognition

## Abstract

**Background:**

Older adults with chronic disease struggle to manage complex medication regimens. Health information technology has the potential to improve medication management, but only if it is based on a thorough understanding of the complexity of medication management workflow as it occurs in natural settings. Prior research reveals that patient work related to medication management is complex, cognitive, and collaborative. Macrocognitive processes are theorized as how people individually and collaboratively think in complex, adaptive, and messy nonlaboratory settings supported by artifacts.

**Objective:**

The objective of this research was to describe and analyze the work of medication management by older adults with heart failure, using a macrocognitive workflow framework.

**Methods:**

We interviewed and observed 61 older patients along with 30 informal caregivers about self-care practices including medication management. Descriptive qualitative content analysis methods were used to develop categories, subcategories, and themes about macrocognitive processes used in medication management workflow.

**Results:**

We identified 5 high-level macrocognitive processes affecting medication management—sensemaking, planning, coordination, monitoring, and decision making—and 15 subprocesses. Data revealed workflow as occurring in a highly collaborative, fragile system of interacting people, artifacts, time, and space. Process breakdowns were common and patients had little support for macrocognitive workflow from current tools.

**Conclusions:**

Macrocognitive processes affected medication management performance. Describing and analyzing this performance produced recommendations for technology supporting collaboration and sensemaking, decision making and problem detection, and planning and implementation.

## Introduction

For older adults with one or more chronic diseases, maintaining health typically requires continual management of complex medication regimens [1,2]. These regimens involve taking multiple drugs, each with complicated names, directions, and purposes, several times a day on differing schedules [3]. Often constrained by age, disease-related cognitive and physical decline, and having to navigate a complex health care system, it is no surprise that many do not take their medications as prescribed [4,5]. Lack of medication adherence is associated with poor outcomes, including increased rates of institutionalization, disability, and death [6-8].

Heart failure is one chronic disease with especially complex medication and lifestyle management components. Heart failure affects 5.7 million US adults and 12% of older adults; it is the leading and fastest-growing cause of death in the United States [9]. Heart failure is characterized by impairment in the heart’s ability to pump and expel body fluid. Treatment involves consistent medication administration to control fluid accumulation and prevent complications [10,11]. Surprisingly, nonadherence to medications has been reported in 40% to 60% of heart failure patients [12]. Emergency room visits, hospitalizations, and the likelihood of survival are related to failing to take heart failure medications as prescribed [13-15].

Interventions to improve medication adherence have primarily involved educating and motivating the patient, with only moderate effects on short-term and little effect on long-term medication adherence [16,17]. Innovative solutions are needed, and there is interest in the potential of consumer-facing health information technology to improve heart failure medication adherence [18-20]. Health information technology (IT) developed for older adults, however, has inconsistently supported their health management needs [21-26]. Older adults using technology for health management report a lack of perceived benefit, a lack of fit to their lifestyle, and that currently available technology is cumbersome and confusing, adding to rather than reducing the effort required to manage their health [27]. According to the principles and international standards for user-centered design, the above problems can be proactively addressed by basing health IT design on an explicit understanding of users, their activities, and their contexts [28-32]. Understanding the actual work health IT is intended to support is the starting point for designing effective technology [33]. Therefore, design of health IT to effectively promote medication adherence in older adults requires a deep understanding of the work activities and work context of medication management [34,35]. We define the concepts of patient work and medication management in Textbox 1.

**Definitions of concepts of patient work and medication management.**

- Medication management is the process of related activities enabling the optimal use of medicines to achieve maximum health benefits with minimal harm for a specific patient [36]. We avoid the term “self-management,” which implies the patient acts alone.
- Patient work is the “exertion of effort and investment of time on the part of patients or family members to produce or accomplish something” [37]. Health-related patient work bears some similarity to paid professional work (eg, assessing symptoms, wound care) but includes unique tasks such as coping with disease progression, scheduling appointments, managing health finances, and preparing diet-appropriate meals [35,38]. Patients also engage in collaborative work, in which either the patient or family member and at least one health care professional are active participants (eg, in-visit communication and shared decision making) [39].

Prior research reveals that the patient work process related to medication management is complex, cognitive, and collaborative, rather than the linear execution of simple, standard tasks. Sensemaking, defined as the deliberate, continuous effort to understand relationships between people, places, and events in order to anticipate their path on which to base actions, is a foundational medication management activity [37] and is essential to chronic disease management [40]. Other medication management processes identified in prior research include tracking, collaborating, ordering, and organizing [38]. In the case of heart failure, some define patients’ self-care (including medication management) as a process of naturalistic decision-making involving situation awareness, mental simulation, and outcome evaluation in the face of uncertainty, ambiguity, and time pressure [39]. Research on health IT functionality has described medication management activities as seeking information, maintaining autonomy, reconciling medications across multiple clinicians [1,41], planning, and creating reminders [42-44]. Nevertheless, these cognitive processes of medication management have not been studied simultaneously in a single group of patients. This has precluded an integrated, systematic categorization and modeling of cognition in medication management in its full complexity. Furthermore, to design effective tools and technologies for older adults with heart failure, it is necessary to understand the unique cognitive workflow of heart failure medication management as it occurs in actual practice.

Our objective is to describe and analyze the work process of medication management by older adults with heart failure, using a macrocognitive workflow framework to adequately capture the complexity of medication management work. Our research framework extends the Workflow Elements Model [45], which portrays workflow as a set of continually evolving and changing processes. Workflow can be planned, routine, and sequential but often emerges based on situational factors and interaction between workflow elements. Those elements are *actions*, performed by *actors* using *artifacts*, producing *outcomes*, supported or constrained by the secondary elements of *context* (ie, physical, social, cultural environments), *timing* (ie, scheduling and coordination), and *aggregation* (ie, interactions, combinations). Our study expanded the model to better operationalize the actions component of the model as a set of macrocognitive processes, such as sensemaking, replanning, coordinating, problem detecting, and deciding [46,47]. Macrocognitive processes are “the collection of cognitive processes that characterize how people think in natural settings” [48]. Macrocognition is explicitly theorized as the type of cognition occurring in complex, adaptive, and messy nonlaboratory settings and can be accomplished by multiple people and supporting artifacts [46]. Thus, combining the Workflow Elements Model with macrocognitive processes facilitates the study of “workflow in the wild” rather than “workflow in a textbook.”

## Methods

During 2012-2014, we performed a study on the self-care of older adults with heart failure. A sample of 61 patients was enrolled in the study and 31 informal caregivers consented to participate in patient interviews, at times multiple per patient. Caregivers often answered questions or added to patients’ answers. Patients and, if present, caregivers were observed during clinic visits and at home and participated in either an extended interview lasting 90-120 minutes or in a short 30-minute interview followed by a longer 90-minute interview. Data from electronic medical records and self-administered standardized patient surveys with a 97% response rate provided additional data. Interviews were semistructured and probed about the actors, artifacts, actions, outcomes, and context of heart failure self-care in general, and of medication management in particular. Interviews were structured on a model parallel to the Workflow Elements Model, namely the Systems Engineering Initiative for Patient Safety (SEIPS) 2.0 model [39], which includes: people; tasks; tools/technologies; social, physical, and organizational context; physical, cognitive, and social processes; and outcomes. A separate subset of questions was asked of each participant, including questions about the perceived efficacy and side effects of medications, medication errors, and medication management tasks such as refills.

Patient participants were aged 65 years or older and lived in a 200-mile radius of Nashville, Tennessee, USA. Half of them were recruited from an outpatient cardiology clinic specializing in heart failure, while the rest comprised discharged patients diagnosed with acute heart failure. Participants (caregivers and patients) provided informed consent and only patients received up to US $65 for participation, to use or split as they wished. The study was approved by the Vanderbilt University Institutional Review Board and Human Research Protection Program. Detailed descriptions of sampling plans and data collection methods are reported elsewhere [38].

Analysis organized findings and major themes into the core elements of the Workflow Elements Model, focusing primarily on the actions (process) element. Within the actions element, data were analyzed according to 5 macrocognitive processes: sensemaking, planning, monitoring, decision making, and coordinating [47,49]. The specific data analysis method was descriptive qualitative content analysis with iterative category development [50]. This method systematically derives trends, patterns, and themes from large amounts of textual data, revealing the underlying meaning [51]. During first-pass structural coding [52], researchers RSM and RJH identified broad passages of data mentioning the management of medications as defined previously. In the second-pass analysis, author RSM assigned initial thematic codes related to broader categories of macrocognitive processes. Definitions of macrocognitive process were based on those established by Patterson and Hoffman [47] and Crandall et al [49]. Next, macrocognitive subprocess categories were iteratively identified using constant comparison [53], after which they were compared to definitions from an extensive review of the macrocognition literature (Table 2), with the final categories adapted to fit macrocognitive processes found in the data. Analysis memos documenting category development decisions were kept throughout this process [50]. Themes within and across categories were noted, for example, describing how macrocognitive processes were related or how a subprocess could break down. Authors RSM, RJH, and KMU met approximately every 2 weeks for a 10-month period to discuss coding and category development; coding exemplars in the form of quotation tables for categories and subcategories was one of the things discussed. Such coding discussions are a proven technique for facilitating analytic convergence among multiple coders [54,55] but in our single-coder arrangement contributed to conceptual clarity and corrections of coding errors.

## Results

### Participants

Table 1 describes patient participant demographic characteristics, caregiver support, and living arrangements.

### Overview

**Figure 1:**
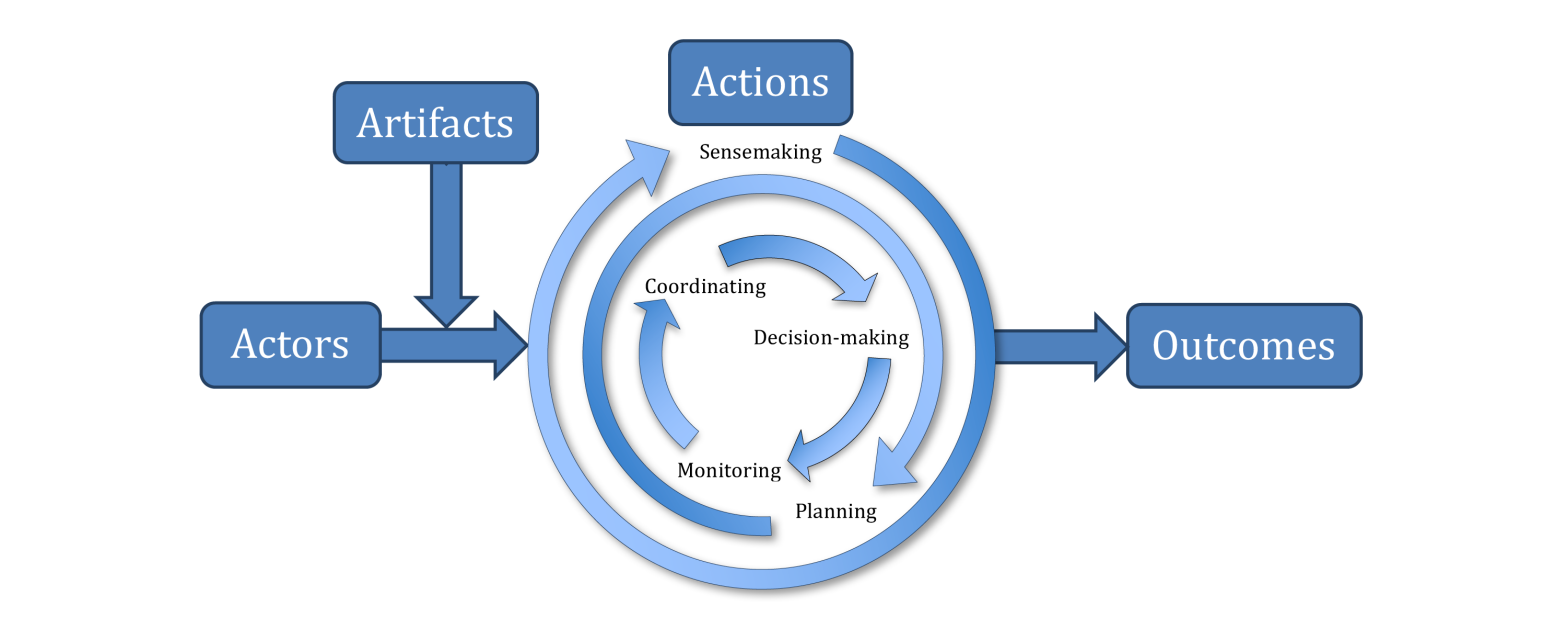
The macrocognitive workflow of medication management (adapted from the Workflow Elements model [45]).

Medication management involved far more than administering pills on time, opening bottles, or binary decision making on whether to take a medication. Behind individual tasks were a host of interacting cognitive processes, promoting a holistic understanding of what patients and caregivers need to do to manage medications in real world situations. Managing medications and the outcomes thereof involved a complex, interacting, and interdependent flow of actors performing actions enabled by artifacts (Figure 1).

Our focus, the actions element of the Workflow Elements Model, and other elements are briefly described in the following sections.

**Table 1:** Patient demographics (N=61).

Demographic variables			% or mean (SD)
Age 65-86 years, mean (SD)			73.31 (6.73)
Male gender, n (%)			31 (51)
White race, n (%)			45 (74)
**Annual income in US $, (n=56), n (%)**			
	<25,000		19 (34)
	25,000-49,000		18 (32)
	50,000-99.999		14 (25)
	≥100,000		5 (9)
**Reported years since heart failure diagnosis (n=52), n (%)**			
	<1		14 (27)
	2-9		24 (46)
	≥10		14 (27)
No. of medications 3-34, mean (SD)			16.9 (5.53)
**Comorbidities^a^**			
	Hyperlipidemia		50 (82)
	Hypertension		55 (90)
	Diabetes Mellitus		37 (60)
**Caregiver support, n (%)**			
	None		32 (52)
	Spouse		18 (30)
	Adult child or children		11 (18)
**Living arrangements, n (%)**			
	Alone		19 (31)
	With spouse		33 (54)
	With sibling		7 (11)
	With adult child or children		1 (2)
	With grandchild		1 (2)
**Other assistance, n (%)**			
	Assisted living		5 (8)
	Home health		7 (11)
Retired, n (%)			55 (90)	

^a^Commonly associated with congestive heart failure, not intended to be a list of all comorbidities of patients in our sample.

### Actors

**Figure 2:**
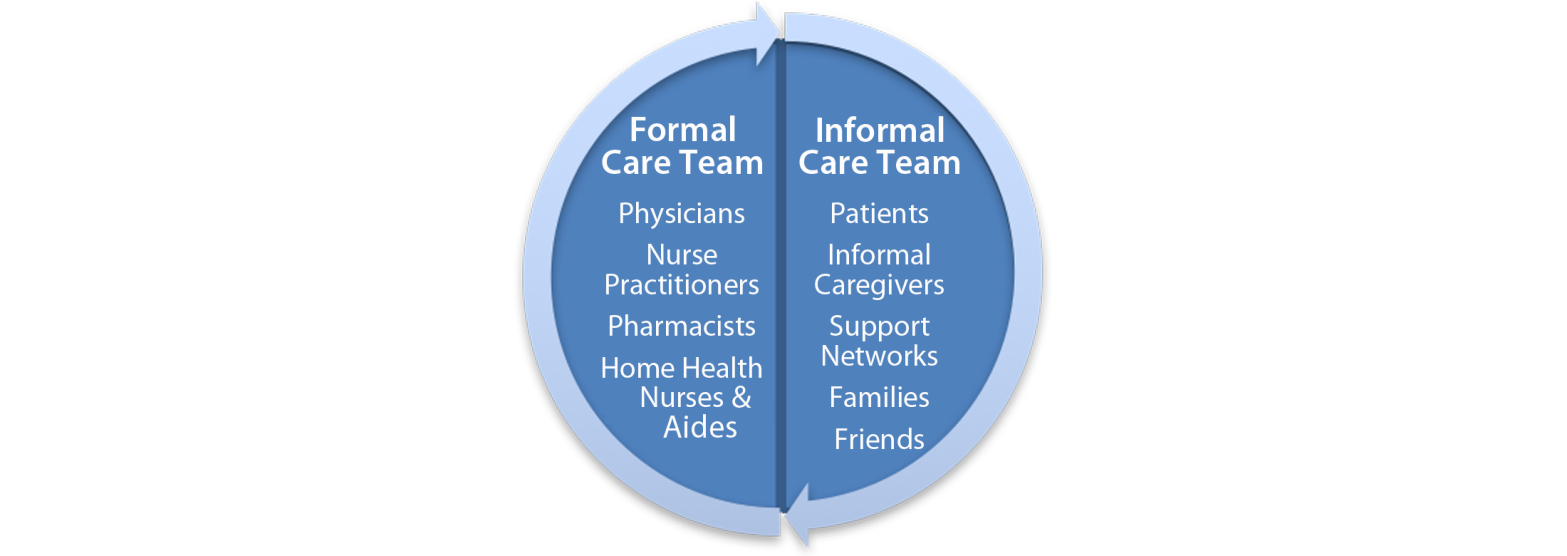
The actors constituting the formal and informal care teams.

A variety of laypersons and health care professionals participated alongside the patient in medication management activities (Figure 2). Informal caregivers, if present, included spouses, adult children, friends, and grandchildren. Their help was dynamic, far-reaching, and varied based on their availability and the needs and desires of the patient. The son of an 85-year-old woman explained how the family administered his mother’s medications: “It started out my sister did it primarily. Then she showed me, and then mom just wanted to do it herself sometimes, but we check.” Assistance sometimes included sharing medications. An 85-year-old man expressed comfort knowing “my sister has some of the same medicine that I take...I can borrow some from there.” Informal team members varied widely in skills, abilities, knowledge, and motivation.

The number of health care professionals comprising the formal team varied with the patient’s condition, comorbidities, and need for home health services. These individuals assisted the patient in a variety of clinical and nonclinical settings. Clinicians who prescribed medications included nurse practitioners specializing in heart failure and physicians with specialties in primary care, cardiology, endocrinology, nephrology, neurology, and pulmonology. Some patients received medication-related assistance in their homes or assisted-living facility from nurses and aides. A 65-year-old patient described not having to leave her home for a blood test to determine the dose of a medication: “It helps me a lot when the home health nurse can come and do my INR (coagulation test) ...and then, she calls that into the Coumadin clinic.” Pharmacists also assisted patients. An 81-year-old patient consulted his pharmacist when his blood pressure was high: “He (pharmacist) said, well now it should’ve gone down, but he says Norvasc is a tricky medicine, it may take it 3 hours to go down, but it will finally go down.”

**Artifacts used by older adults with heart failure.**

(1) Patients and informal caregivers used tools for monitoring and measurement (eg, blood pressure cuffs, scales), tracking and communication (eg, vital sign logs, medication lists, online patient portals), organizing administration (eg, pill organizers, baskets), and gathering information (eg, Internet, books, brochures). Many patients (37/61, 61%) used pill organizers to decrease the effort of managing multiple medications and reduce the possibility of error. Some patients and informal caregivers used an online patient portal (20/61,33%) provided by their medical center and found the portal useful for communicating with health care professionals about refills and other needs.

(2) These tools did not always adequately support medication management activities. For example, some patients adapted medication lists received from the clinic. The son of an 84-year-old patient explained why his mother used an old medication list:

*   And sometimes there’s been a print out from them (clinic) around, but somehow or another this is just the one we have been using. Particularly    because it will also help by telling me what it’s for (referring to hand-written annotations on purpose of each medication).*

(3) Personal devices including blood pressure cuffs used by some patients were originally designed for clinical use and patients and informal caregivers did not always understand the meaning of the raw numerical output. For example, a 68-year-old patient described his blood pressure reading to his nurse practitioner: “Well, let's see, the other night I was sitting there resting and it was good. I believe it checked it, I checked it, it was, uh, 198 over 136.”

(4) Multiple medication representations (eg, medications, prescription labels, numerous medication lists, electronic health record lists) were difficult to reconcile across care settings. For example, a 65-year-old patient could not remember the name of a prescribed medication, but knew its timing and appearance: “I have to take it twice a day, it’s supposed to be three times, I take it twice a day. It’s orange and kind of brown.”

### Artifacts

Artifacts—tools and technologies—facilitated patients’ medication management. We have previously described the artifacts used by heart failure patients in this study [56]. Textbox 2 summarizes these findings [56].

### Actions

For ease of presentation, we describe medication management actions in categories of discrete macrocognitive processes in Table 2. However, these processes interacted, overlapped, and were alternatively concurrent and sequential. For instance, when a patient gathered information about a medication (a subprocess of sensemaking), decision-making and planning were likely also taking place. Table 2 defines the macrocognitive processes and subprocesses reported in this study.

#### Sensemaking

Sensemaking actions described by participants were retrospective, deliberate processes that integrated new information into existing understanding to guide future action. Sensemaking processes were foundational, contributing to all macrocognitive processes.

Due to the continuous flux in patients’ health and medication regimens, punctuated by various health-related events (eg, hospitalization, new prescription), participants perpetually searched for meaning and causal explanations by gathering information, adapting mental models, and storybuilding.

Information gathering occurred across actors, locations, and time. During clinical visits, most of the questions from observed participants were about verifying or executing an existing medication plan. They asked questions such as: “How many do I take? *”* [65-year-old male], “You sent her refill in, didn't you?” [daughter of 74-year-old female], and “Can I have a dental exam (while on an anticoagulant)?” [65-year-old male]. These questions implied a concern for “what do I do” more than “why do I do it.” Many patients (46/61, 75%) also gathered medication information from sources outside the clinical setting (Table 3). Reasons for gathering additional information included (1) a new diagnosis requiring medications, (2) an upcoming procedure, (3) a change in the medication regimen, (4) questioning the validity of medication choices made by clinicians, and (5) uncertainty or anxiety. Participants commonly gathered information from laypersons such as family, friends, or support groups. They sometimes shared this social network-sourced information with clinicians. A 65-year-old patient suggested to his physician: “So, one of my friends said well maybe you just need a, a pap, what do you call it? Pa-, Paxil, is it?” Participants who mentioned Internet or television information viewed it as valid and authoritative but had difficulty filtering and prioritizing it.

**Table 2:** Medication management process and subprocess definitions.

Process	Subprocess	Definition
**Sensemaking**		Deliberate, retrospective efforts to understand and explain events typically triggered by a change [57].
	Information gathering	Exploratory activities to “gather, differentiate, interpret, evaluate, and aggregate” information from sources [58].
	Adapting mental models	Reframing internal representations (how things work, mechanisms) on which to base future actions and expectations [59,60].
	Storybuilding	The process of constructing narratives (stories, scripts, schema) to infer how a current situation might have evolved from an earlier state [61].
**Planning**		Generating and adapting methods for action to transform current state into desired future state [49].
	Generating plans of action	Generating options for methods by balancing available resources and existing constraints to achieve a specific goal [62].
	Adapting plans	Responding to changes in goals from a variety of sources such as peers, constraints, opportunities, events, or changes in anticipated plan trajectories [47].
	Anticipatory thinking	Preparing to respond to constraints, contingencies, and opportunities that could be encountered while implementing a plan [62,63].
**Monitoring**		Maintaining awareness of system state; to observe and check the progress or quality of (something) over a period of time; keep under systematic review [64].
	Problem detection	Noticing when events may be taking an unexpected direction [47].
	Tracking	A control process that follows the course or progress of something to keep the system within safe and acceptable levels of performance [65].
**Decision making**		Commitment to one or more options or actions [47,66].
	Applying rules	Using a prescribed, explicit, and understood regulation as a guide for conduct or action [64].
	Pattern matching	Matching the circumstances of the present situation to similar events and clusters of cues from the past [63].
	Mental simulation	Imagining how a decision will play out [67].
	Making trade-offs	Losing one quality or aspect of something in return for gaining another quality or aspect [68].
**Coordinating**		Managing interdependencies across members of a team with overlapping, common, and interacting activities, roles, and possible conflicting goals [47,69].
	Reconciling information	The process of bringing information or understanding into agreement (ie, maintaining common ground) [69].
	Managing interdependencies	Managing the mutual reliance and dependencies between elements of a system [69].
	Negotiating	Coordinating competing roles, goals and plans in the “give and take” process by which team members agree on a common issue or solution [70].

**Table 3:** Information sources outside of the clinical setting (n=61).

Information source	%	Information type
Medical Center Portal (n=20)	33	laboratory tests, diagnostic tests, clinical summaries, lists of current medication regimen
Internet (n=25)	41	websites with health, disease, and medication information
Television (n=5)	17	commercials, TV shows (eg, Dr. Oz)
Educational print materials (n=14)	23	medical books, medical brochures, information booklets
Educational classes (n=2)	3	organized diabetes, heart failure instruction
Prescription inserts (n=6)	10	medication indications, dosing, side effects, special instructions
Family, friends, support groups (n=27)	44	shared personal advice, experience, knowledge

Participants synthesized gathered information with previous experiences and current knowledge by adapting mental models or their personal understanding of “how things work.” To illustrate, a 75-year-old patient revealed not taking her medications because she perceived they had no effects on her health, and did not like taking “so many” medications. She explained that after a hospitalization and conversation with her physician, she revised her mental model to view medications as similar to vitamins: “Medication is a form of preparation, you know, and builds your system up to fight off what may come in the future.” After this mental model revision and a reduction in the number of daily medications prescribed by her physician, she subsequently began to take her medications regularly.

At times, participants developed inaccurate mental models, especially regarding functional or causal relationships between body systems, medications, and health events. A 75-year-old female patient contended, “I don’t have no heart failure medicine. I only have blood pressure medicine.” Several participants had difficulty connecting fluid retention to heart functioning. An 85-year-old female patient elaborated, “I don’t think it’s (fluid) in the ankles or the hands or anything like that. I think it’s the fluid in the heart area that would make the heart beat less.”

Table 4 gives examples of participants’ descriptions of causal factors contributing to past health events.

**Table 4:** Example causes of health events described by patients and informal caregivers.

Cause	Quotes
Prescribing decisions	The rejection (heart transplant) and it was due to their neglecting, negligence of not resuming my appropriate therapeutic level of Procrit, my medication. [68-year-old male patient] They gave him an overdose of it (Lasix). [wife of 72-year-old patient on why her husband experienced kidney failure] My hair has fell out because they took me off my medicine. [65-year-old female patient]
Medications	Yeah, that’s (medication) what made me mean. I kicked a t-, a tray out of the nurse’s hands and stuff like that when I was in the, in the rehab. [78-year-old male patient] Well, all the other times, you know, I'd never had it (diabetes)... Some of the medication that they put me on would cause high sugar. [68-year-old male patient]
Procedures	Okay. Yeah, um, I think most of my health problems came after an open-heart operation, mitral valve repair in late 2001. [81-year-old male patient] Some of his memory problems...but he was put to sleep four times in two months and that really isn’t very good. [wife of 81-year-old male patient]
Genetics	It’s certain things and this is a genetical (sic) thing with a black man’s diet and a white man’s diet. See, uh, we grew up on pork that’s the worst meat you can eat. Pork, half dog, half rat, half, and they eat anything, you understand? [67-year-old male patient explaining the cause of his high blood pressure]
Comorbidities	I think it (stroke) take a toll on my heart... That is why I have a pacemaker. [79-year-old male patient]
Symptoms	So I think all that pain and all may have caused heart trouble. I don't know. [74-year-old male patient]
Environment	That portion of when I look back now was a lot of just losing my breath, shortness of breath and all, came from the room fresheners. [68-year-old male patient]

Storybuilding was a subprocess that enabled the creating and updating of mental models as well as organizing information and communicating one’s mental model to others. A 69-year-old patient retold the story behind her pacemaker insertion:

I was seeing a doctor and he had increased my medicine, Coreg, and the more he increased it the less my heart functioned so that’s when they decided they had to...so I came back, I moved my mother, came back down here and, um, uh a doctor put in my pacemaker.

In summary, patient and informal caregiver sensemaking (1) combined information gathered from multiple sources including sources outside health care settings and past experience, (2) developed causal models for health events, and (3) produced new or revised mental models often expressed in personal stories.

#### Planning

Planning was the practical, prospective translation of instructions into implementable actions under known constraints, with the goal of achieving a desired future state. Generating plans of action provided the “how” of performing generic instructions such as “take Lasix three times a day” in practice.

Participants expressed planning as an ambiguous process not well supported by their formal care team. A 74-year-old male patient described the lack of guidance for planning: “There’s, there’s not a, you know there’s not a magic list of instructions that they lay out.” A recently discharged 65-year-old patient similarly conveyed the lack of guidance after her hospitalization: “When you go home, you’re kinda on your own. You’re kinda flyin by the seat of your britches.”

As participants recognized changes in symptoms, medication regimen, available resources, and existing constraints, they were continually adapting plans. To exemplify, a 66-year-old patient explained how mixing up 2 look-a-like medications resulted in an adverse drug reaction; consequently, he planned to break a newly prescribed medication in half to distinguish it from other pills. As in this case, action plans often arose from new awareness of constraints (look-a-like medications) based on feedback from implementing a prior plan (adverse drug event). After experiencing severe shortness of breath that led to a hospitalization, an 84-year-old patient decided weight was not a sensitive indicator in detecting fluid retention. He instead planned to use a pulse oximeter to dose his conditional diuretic. He observed nurses using the device in the hospital and saw other patients with the device in the clinic waiting room. Although not directed by his clinicians to use the device, he explained his rationale:

No, no one told me, but I know what happens when you don’t have enough oxygen... I don't take any chances. When my oxygen gets down and doesn't come above 96, 95 or 96, I, I consider that a, uh, uh, a push a go button to do something.

This plan, however, was potentially unsafe and may have resulted in the diuretic overuse and resultant kidney damage.

Planning and replanning often created new routines and leveraged known resources such as pillboxes [56] or a patient’s “self-care workspace” [71].

You can put the daily dose in each (pillbox compartment) in advance so you don’t overlook it. Because trying to open half a dozen containers twice a day, is impossible

81-year-old male patient

So it’s all right there when he sits at the table where he can get to everything and that makes a difference too. You know that reminds him to do it.

Daughter of 80-year-old patient

Anticipatory thinking aided planning; projecting into the future possible consequences, constraints, and opportunities that might be encountered when implementing a plan. A 70-year-old patient explained a strategy he created in anticipation of forgetting whether he took his insulin:

I’ve le- got a system for that now too anyway... I keep all, it takes ten syringes out of the little bag and I put them in, with the rest of my in-, with my insulin and stuff and if, if I’ve got an even amount that means I haven't taken the morning one, but if I s-, if later on if I’ve got an odd amount it means I didn’t take that evening medicine.

Participants placed high value on planning as a method to cope with uncertainty and anxiety. A 67-year- old patient emphasized the importance of filling pillboxes weekly to assure she did not forget to take her medications: “I don’t, I don’t forget that. That’s my lifeline. How do you forget your lifeline?” This and other observations illustrate planning as a method of control over complex medication management requirements.

#### Monitoring

Monitoring involved what participants called “listening” or “watching” for changes. Endsley [72] and other researchers have previously described this concept as maintaining situation awareness, defined as perceiving the current state, interpreting its meaning, and projecting the future. Problem detection occurred when a participant noticed something wrong with the current state whereas tracking occurred as people followed data over time to identify patterns and trends indicating a potential future problem. To illustrate the distinction, noticing that a medication bottle was empty involved problem detection, while documenting medication refill dates involved tracking.

Problem detection required “noticing” an anomaly, yet many participants described difficulty in distinguishing between symptoms and the effects of medications. A 68-year-old patient recounted an instance of this confusion when she forgot to take a morning medication: “I really didn’t feel you know that bad. Um, of course it could have been one of those days I was feeling not that good anyway.” Not understanding the expected effects of medications compounded ambiguity, as did the lack of perceivable problem cues. Patients developed their own cues based on experience. Many patients (26/61, 43%) created a personal “sign” of fluid retention. A 68-year-old woman described hers: “I knew the signs of my congestive heart failure, and which mine is, I might get a little smother some and my irregular heartbeat and a little bit of discomfort in my chest.” An 83-year-old retired physician shared his: “(It is) how much trouble I have getting in my pickup truck. If I'm short of breath after I do that, then, I know that I'm in failure.”

Detecting medication administration problems such as forgetting or mixing up medications was important but unlike symptom detection, did not benefit from personal warning signs. Some participants recalled instances when they forgot or took the wrong medication and were not aware until the next administration time. A 68-year-old male patient recounted: “I opened up the little box for my morning pills, the (bedtime) pills were still in there.” Some participants questioned the appropriateness of medication prescriptions and went to the Internet to “follow behind” and “check if it’s right” or validated with other clinicians to verify if the right medication was prescribed.

Compared with problem detection, tracking was a longer-term, forward-looking function. Some information that participants tracked was very specific. One patient kept a list of medications he could not tolerate to assure an unknowing clinician did not prescribe them in the future. Another patient tracked refill information (eg, prescription number, ordering clinician, refill date) in a self-made chart. Two patients documented when they administered an as-needed diuretic on their vital sign logs to prevent over-administering the medication. One patient tracked the cost of her medications at various pharmacies and switched pharmacies to avoid going into the “doughnut hole,” a maximum yearly limit imposed by the Medicare insurance plan. Patients and caregivers also tracked information in a less purposeful manner or “just in case” it was needed. Some stored all the documents they received from their clinicians or hospital discharges. Information was also tracked as stories, adding to either an individual or shared narrative, as illustrated by the following piecing together of a medication misadventure by a 74-year-old patient, her husband, and a nurse practitioner (NP):

Husband: Well now, they give her, I can't even think. He give her one, one time, but that put her back in the hospital... It, it was just a little pill, but...

Patient: I lost my arms and legs, the use of 'em. I don't know how many times he's had to get up and pick me up. I, it was once a week.

NP: I think I remember that.

Patient: What doctor was it? Do you remember?

Husband: That one that shocked her heart... It was just four milligram. We took it once a week, but man, it put her down.

Some participants assumed the electronic health record tracked their medical information and therefore they did not need to track this information themselves. A 65-year-old patient did not bring a copy of her medication list or the medications themselves to her cardiology appointment and dismissed the need: “They always just get it off there (electronic health record). Nothing has changed.” However, during the appointment several medication discrepancies were discovered.

#### Decision Making

Decision-making processes resulted in a variety of decisions, including calling a clinician, taking or skipping a medication, or modifying behavior (eg, diet). Table 5 provides examples of how participants made decisions involving potential fluid retention, indicated by swelling or sudden weight gain. Some medication management problems had solutions prespecified by a clinician and could be solved by applying rules for the appropriate situation. Some patients (12/61, 20%) had a clinician-provided rule to take an as-needed diuretic when their weight exceeded a threshold value. These rules were helpful but not all patients received rules and some had rules they did not follow. Participants also often established their own rules and decision-making criteria based on their own or others’ experiences. For example, a patient did not begin taking a medication his primary care physician prescribed until he spoke to his cardiologist; this rule stemmed from a negative experience with a nonspecialist prescribing cardiac medications in the past.

**Table 5:** Medication decision making for fluid retention.

Process	Decision	Quote
Applying rules	Call clinician	I mean I have instructions from (clinician) if your weight goes up this much in two or three days call me. [74-year-old male patient]
Gathering information	Delay	And it was, it (blood pressure) was an hour earlier, the difference in a hour uh so I take it again if it was, seemed to be off. [80-year-old male patient]
Pattern matching	Seek assistance	So, I monitor that (weight) fairly carefully. If it goes up, I usually call and say, "What do I do now, daddy?" [80-year-old male patient]
	Use familiar action	I just take an aspirin (for shortness of breath), or I take some Tylenol. [83-year-old female patient]
	Do only as instructed	They said to check it (blood oxygenation) and if it’s a certain level then it’s okay. But then when it’s not, you know they said let, you know write it down. [wife of 70-year-old patient]
	Use a familiar action for a similar symptom	I used to have childhood asthma, occasionally I’ll wake up at night with a slightly asthmatic tight feeling and sort of I’ll walk it off. [81-year-old male patient, describing his response to heart failure symptoms]
Making trade-offs	Prioritize medication goals	I just stayed home, you know. There was no (bladder) control at all. [80-year-old male patient]
	Prioritize personal goals	So I didn’t take it (medication) then for several days in a week or two-week time... I didn’t want to be, uh, be stopping on the road every fifteen minutes. [67-year-old male patient]

Participants sometimes utilized pattern matching. The husband of a 65-year-old patient explained how his wife (wrongly) matched her usual solution for coughing to her shortness of breath from fluid retention: “I’ll tell you what she does when she had, is having a problem breathing... She’s got on these menthol cough drops... and sometimes she’ll take up to ten or eleven of them.” Participants also used mental simulation in making decisions. An 83-year-old man responsible for the care of his debilitated wife did not contact a clinician when he experienced shortness of breath because he imagined it would result in hospitalization and consequently leave his wife unattended.

Making trade-offs was a decision making subprocess that occurred when participants confronted conflicting goals and unclear solutions.

I ended up having blood in the urine and this, this, well this creates a problem so, you know, you talk to them and they say drink lots of water, a lot of liquids, you know. Well I drink lots of water, a lot of liquids and what happened is it didn’t stop bleeding right away but it sure filled me up with water. I couldn’t breathe and I mean I had a heck of a time.

74-year-old patient

Participant trade-offs sometimes involved going against medical advice. A compromised kidney function required the physician to discontinue a 74-year-old patient’s gout medication. During an acute gout attack, however, she took the discontinued medication, “They (physicians) took my gout medicine away from me and I told (husband), I said you just get that right back... I said if you don’t want to give it to me, I’ll take it from myself and so, so I did.”

#### Coordinating

Due to the distributed nature of the patient care team, coordinating information and activities across locations, actors, artifacts, and time required continual effort. Coordinating enabled and constrained other macrocognitive processes. Reconciling information brought actors and artifacts into agreement by updating one another and identifying discrepancies. For example, an 85-year-old patient described reconciling new medication information with his informal caregiver and a medication artifact: “(when) I know they’ve changed my prescription, I make a note and call her (daughter) and tell her so she put it on her list and I write on my top (of pill bottle).” During clinic visits, medical assistants reconciled the electronic health record medication list with the patient’s paper list, prescription bottles, or memory. Discrepancies were common and not all information was reconciled or shared. An 81-year-old patient stopped taking medications when he traveled but “never discussed it” with his physician. Coordination breakdowns at times stemmed from not reconciling clinician provided information with a patient’s understanding. A good illustration was a 65-year-old man being unaware he recently suffered a heart attack based on information he received at the hospital: “It (heart attack diagnosis) was a surprise cause it, they (just) told me, they told me my enzymes was elevated.”

Coordination was also accomplished by managing interdependencies (actions and information) between care team members across time and space. Timing of clinical appointments often depended on the availability of a family member to drive. A pending surgical procedure required an 81-year-old patient to inquire with his cardiologist about when to discontinue an anticoagulant: “They (surgeon) want to know what I need to do about getting the okay to stop the Coumadin.” Participants did not always manage interdependencies effectively. There were many examples of communication breakdowns between care team members. In one example, a 72-year-old woman received the wrong medication from the pharmacy after a hospital discharge. Her frustrated daughter explained, “She (pharmacist) said well they faxed it in, but you still got some on the other one so they ain’t never filled that new prescription that he (physician) called.”

Coordination also required negotiating roles, treatment plans, and medication goals. A simple example of role negotiation was the wife of a 74-year-old patient informing the cardiologist she did not need him to refill prescriptions: “I’ll just get him (primary care physician) to do all of his prescriptions.” Roles were also dynamically negotiated between patients and family members. When asked who was responsible for administering her medications, an 85-year-old patient stated, “Well everybody is really. If sometimes, you know I usually get it (medications) myself, but sometimes I’m just so tired I’ll ask (for help).” Patients negotiated medication regimens with their clinicians. A patient who did not like swallowing pills negotiated with her cardiologist to decrease the number of daily pills from 8 to 4. In contrast, some participants omitted, decreased, or increased medication doses without coordinating or communicating with health care professionals. The son of a 79-year-old patient described the medication “tinkering” practice of his father: “He likes to play doctor for himself you know.”

### Outcomes

**Figure 3:**
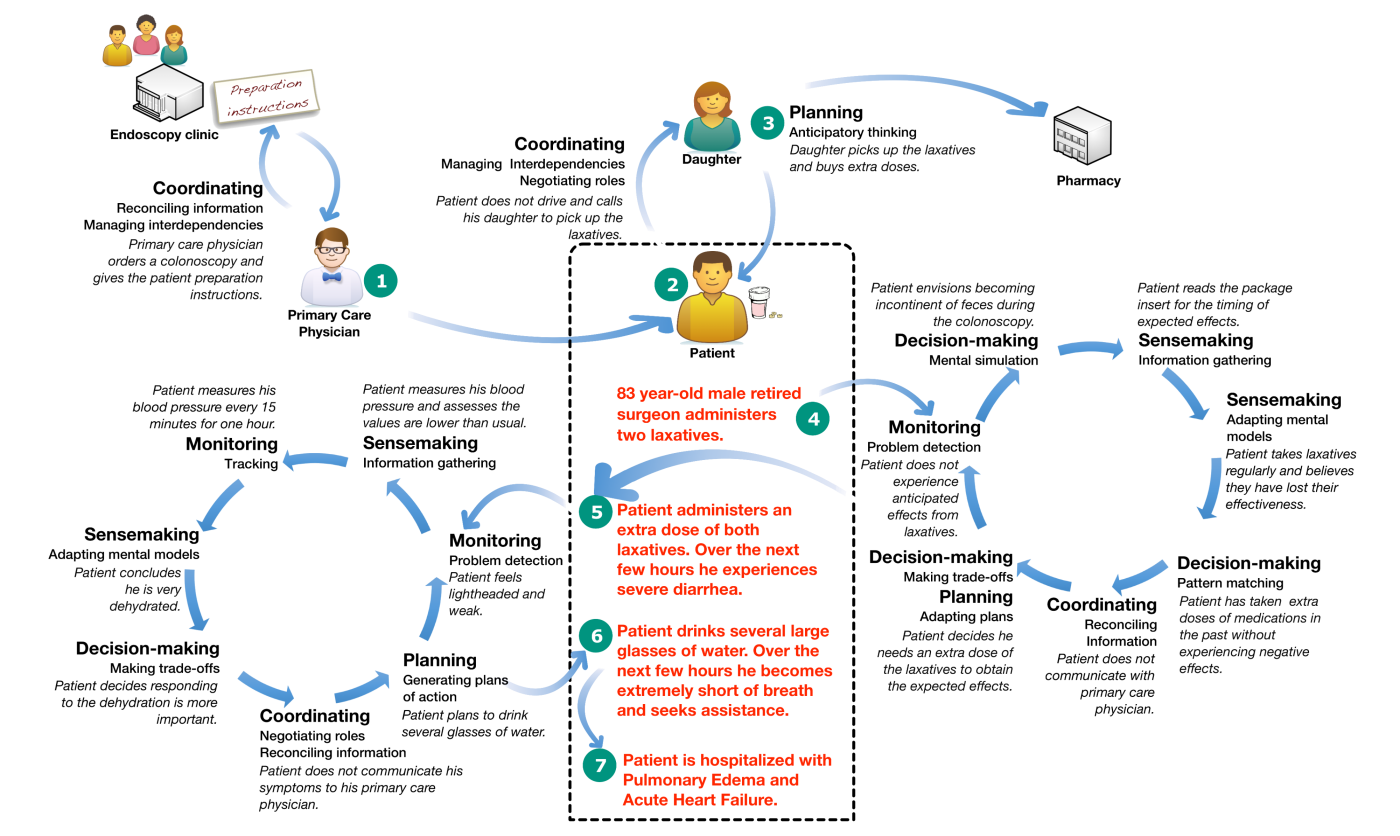
Patient macrocognitive workflow scenario.

The interactions between macrocognitive processes and other elements of the medication management system produced successful and unsuccessful outcomes. Textbox 3 and Figure 3 present a patient scenario illustrating macrocognitive processes and their relationships to outcomes based on one participant narrative.

**Scenario of medication management outcomes.**

An 83-year-old retired surgeon is scheduled for a routine colonoscopy. Written instructions from the endoscopy clinic are given to him by his primary care physician and instruct him to administer a combination of laxatives the day before the procedure.

The patient self-administered the laxatives in the morning the day before the procedure. He was anxious about the colonoscopy because he occasionally was incontinent of feces. He did not want to have an accident during the procedure.

Hours after the administration of the laxative, he perceived no effect. He decided to administer an extra dose of the laxatives. Later he experienced a large amount of diarrhea and became lightheaded. He perceived himself to be dehydrated and drank several large glasses of water.

Several hours after drinking the water, he became extremely short of breath. He called for assistance from the assisted-living facility he lived in. When she saw the patient, the medical assistant immediately called an ambulance. The patient was admitted to the hospital for pulmonary edema and acute heart failure.

## Discussion

### Expanded Framework

Expanding the scope and frame of patient medication management uncovered insights into previously unexplored cognitive processes underlying performance. Broadening this lens confirmed the complex, cognitive, and collaborative nature of medication management workflow suggested by previous research. This analysis also provided new insights and implications for design of medication management tools and technologies, summarized in Table 6.

Examining processes at a level above individual microcognition allowed for a theoretical expansion of the actions element of Workflow Elements Model (Figure 1). A limitation of that and other workflow and work system models [45,73,74] has been their vague depiction of process (eg, care vs noncare; cognitive, physical, or social-behavioral). Here, actions generically called “cognitive processes” in the past were systematically broken down into distinct functional processes and subprocesses.

Applying the expanded framework to heart failure medication management, we found that these cognitive processes were collaborative, with patients, informal caregivers, and clinicians all serving key roles in care [1,75,76]. Such findings further blur the lines between what is considered patient work versus the work of health professionals, especially as new technologies support patients in carrying out health work previously performed only by health professionals. Researchers now insist that patients and professionals are coproducers of care [77] and perform collaborative patient-professional work [39]. However, we found here and elsewhere [56] that patients and informal caregivers lacked the tools to support collaborative workflow around medication management both within households and across other settings (patient’s home, caregiver’s home, clinic). In addition, patients were not always willing to collaborate with formal caregivers and withheld information or made critical decisions without conferring with them. Openness to enhanced collaboration and communication will require a paradigm shift in the minds of formal and informal team members [77].

Based on the present analysis and prior research, we discuss 3 areas in dire need of well-designed technology: collaboration enables sensemaking, problem detection precedes decision-making, and planning requires implementation. Table 6 summarizes specific recommendations for technology supporting effective macrocognitive workflow during medication management, based on our findings.

**Table 6:** Summary of findings and recommendations for design.

Findings		Recommendations for design
**Collaboration and Sensemaking**		
	Patients or informal caregivers lacked the tools to support the collaborative workflow of medication management.	Design technology with shared access to all members of the care team to promote information sharing and reconciliation. Design technology to support mediated synchronous and asynchronous opportunities for interactions (eg, telehealth technologies, text messaging, email, patient portals). Use structured, automated detection and record keeping of events (eg, prescriptions) to facilitate reconciliation across care settings.
	Patient or informal caregiver mental models were inconsistently shared with health care professionals.	Design structured tools to elicit patient/informal caregiver sensemaking of information and events during formal or informal team interactions. Support for the joint creation of explicit representations of “how things work” to support accurate team sensemaking.
	Patients or informal caregivers struggled to synthesize large amounts of information and translate into actions.	Technology that supports the retrieval and visualization of information from multiple sources into meaningful displays of information. Personalized shared information dashboards editable by all team members.
**Decision Making and Problem Detection**		
	Patients or informal caregivers struggled with decision-making	Design decision-support tools for use by patients and informal caregivers in the home setting (eg, clinical decision rules).
	Patients or informal caregivers value the experiences and behavior of others for decision-making.	Support access through social media to heart failure support groups that include formal and informal team members for sharing stories, information, tips and tricks (eg. PatientsLikeMe). Support access to individuals who can serve as model exemplars, for example, through discussion forums or lay coaching.
	Patients or informal caregivers struggled to detect symptom and medication effect cues.	Collect or use available data (eg, from cardiovascular implantable electronic devices, wearables, smartphone sensing, motion sensors) to automate cue detection or inform patients of the need to be vigilant for cues.
	Patients or informal caregivers relied on electronic health records (EHR) for medical and medication history tracking.	Automate tracking to the extent possible, to counteract cumulative difficulty of tracking. Provide easy access to EHR information or a shared historical health record. Encourage EHR screen sharing during clinic visits.
**Planning and implementation**		
	Patient or informal caregivers lack support for planning and implementation of medication regimens into the context of their own lives.	Support for structured tools to facilitate collaborative medication planning (eg, MedTable [78]) and strategy development. Use projection and simulation to help compare and validate plans. Offer planning tools for a variety of crises and other eventualities (eg, Plan Your Lifespan [79]).

### Support for Collaboration and Sensemaking

Coordination is the core of successful team performance [80] and “wraps” around other macrocognitive processes [47]. Sharing information towards the goal of establishing mutual understandings is a characteristic of high-performing teams [70,81,82]. Multiple comorbidities add to complexity and increase coordination requirements and the data to consider for sensemaking. With growing access to digital information, we found that patients gathered a large amount of data from multiple sources but struggled to synthesize them and translate data to actions. We also identified unidirectional information flow, with patients gathering but not always sharing information, or not sharing it clearly. This led to incongruous mental models between patients and others, with minimal opportunity for making corrections. Our analysis demonstrates that the emerging role of the patient as actor can create silos of information and few guidelines for information sharing. IT can support collaborative information management towards the development of shared understanding and better coordination.

### Support for Decision-Making and Problem Detection

While the majority of work related to clinical decision-support has focused on clinicians in professional settings, our study provided clear evidence that decision-support tools for patients and informal caregivers to use in home contexts are needed. Our results demonstrate that laypeople often make decisions based on their previous experiences and not by comparing options in a risk or benefit type analysis, in agreement with research in other domains [49,83]. Mental simulation, situation awareness, and problem detection were crucial processes enabling decision-making about responding to symptoms. However, as with prior work, it was not clear whether these processes were effectively performed by everyone or only by a subset of patient “experts” [84,85]. Participants also made decisions by modeling the behavior of others, suggesting that technology could help connect patients to individuals who can serve as model exemplars, for example, through discussion forums or lay coaching. Participants also indicated a clear desire for support in judging the appropriateness of decisions made by clinicians.

### Support for Planning and Implementation

Implementing the medication regimen in a patient’s specific life context is challenging. Others have reported that heart failure patients knew “what” to do but struggled with “how” to implement the medication regimen into their daily lives [86]. Having identified the patterns of patients’ planning and execution of medication management in their natural context, we note several implications for technology design in Table 6. In particular, we stress on technology to help patients with 3 key areas of work: develop and strengthen daily routines, plan specific behaviors (eg, using goal setting methods), and compare different implementations of the same general plan (eg, taking medications upon waking vs with breakfast).

### Limitations

The analyzed research interviews had a broad scope of heart failure self-care, including specific questions about medication management. This breadth made it difficult to thoroughly examine medication management for an individual participant but patterns emerged when examining data across participants. The sample was limited to individuals in one region, with many receiving care at the same US academic medical center. This study did not collect data structured enough to develop quantitative workflow models capable of producing state transition probabilities, that is, the flow from one action to another. Finally, observation data were limited compared with interview data. A recent publication suggests the various methods that can be used to more rigorously study patient work phenomena such as medication management workflow [87], and how future work could incorporate additional methodologies. A single coder assumed primary responsibility for codebook development and application, due to resource limitations and institutional expectations of dissertation research projects. All the authors extensively discussed codebook development and used throughout the research, with the lead author presenting multiple examples of how codes were developed, underlying data, and rationale behind coding decisions to coauthors. Although every effort was made to address potential concerns about internal validity of the codebook through extensive and repeated discussions, the primary single coder approach remains a potential limitation of the analysis process. Involving multiple coders in the analysis process could strengthen future analyses.

### Areas for New Research

This study highlighted important new areas of inquiry previously unexplored in patient medication adherence and management research. The collaborative, distributed nature of medication management calls for the application of team models and theories to the understanding of health management behavior. Improving knowledge building, knowledge transfer, and mental models sharing is a promising focus for interventions and technology design. More research is also needed in the area of patient expertise, how expertise is expressed in patient work, and how tacit knowledge develops in individuals and communities through information sharing and experience. Additional research is warranted into assessing the workload associated with cognitive work such as medication management, including better measures of cognitive demands, cognitive resources, and the balance of the two. Of great interest is the notion of articulation work, or the work needed to ensure processes such as medication management can be effectively performed. Articulation work such as managing one’s health insurance and finances to maintain a supply of medication is often “invisible” and under investigated, but a necessary component from a macrocognitive perspective. More research is needed on how to integrate new technology with existing well-functioning artifacts and practices. There is a need for further research using ethnographic methods, cognitive task analysis, and other techniques adaptable to study the work of patients. Methods such as experience sampling methodology or day reconstruction method are needed to understand cognitive work contemporaneously without disrupting patients’ lives, but these methods have their challenges as well, including variability in the depth and accuracy of collected data.

## Abbreviations

IT: information technology
NP: nurse practitioner
SEIPS: Systems Engineering Initiative for Patient Safety
